# Genetic and phenotypic heterogeneity in early neurodevelopmental traits in the Norwegian Mother, Father and Child Cohort Study

**DOI:** 10.1186/s13229-024-00599-0

**Published:** 2024-06-07

**Authors:** Laura Hegemann, Elizabeth C. Corfield, Adrian Dahl Askelund, Andrea G. Allegrini, Ragna Bugge Askeland, Angelica Ronald, Helga Ask, Beate St Pourcain, Ole A. Andreassen, Laurie J. Hannigan, Alexandra Havdahl

**Affiliations:** 1 PsychGen Centre for Genetic Epidemiology and Mental Health, Norwegian Institute of Public Health, Oslo, Norway; 2 Nic Waals Institute, Lovisenberg Diaconal Hospital, Oslo, Norway; 3 Department of Psychology, University of Oslo, Oslo, Norway; 4 Division of Psychology & Language Sciences, Department of Clinical, Educational & Health Psychology, Faculty of Brain Sciences, University College London, London, UK; 5 Social, Genetic and Developmental Psychiatry Centre, Institute of Psychiatry, Psychology & Neuroscience, King’s College London, London, UK; 6 Division of Mental Health and Addiction, Oslo University Hospital, Oslo, Norway; 7 School of Psychology, Faculty of Health and Medical Sciences, University of Surrey, Guildford, UK; 8 PROMENTA Research Centre,Department of Psychology, University of Oslo, Oslo, Norway; 9 Language and Genetics Department, Max Planck Institute for Psycholinguistics, Nijmegen, The Netherlands; 10 MRC Integrative Epidemiology Unit (IEU), University of Bristol, Bristol, UK; 11 Donders Institute for Brain, Cognition and Behaviour, Radboud University, Nijmegen, The Netherlands; 12 Institute of Clinical Medicine, University of Oslo, Oslo, Norway

## Abstract

**Background:**

Autism and different neurodevelopmental conditions frequently co-occur, as do their symptoms at sub-diagnostic threshold levels. Overlapping traits and shared genetic liability are potential explanations.

**Methods:**

In the population-based Norwegian Mother, Father, and Child Cohort study (MoBa), we leverage item-level data to explore the phenotypic factor structure and genetic architecture underlying neurodevelopmental traits at age 3 years (N = 41,708–58,630) using maternal reports on 76 items assessing children’s motor and language development, social functioning, communication, attention, activity regulation, and flexibility of behaviors and interests.

**Results:**

We identified 11 latent factors at the phenotypic level. These factors showed associations with diagnoses of autism and other neurodevelopmental conditions. Most shared genetic liabilities with autism, ADHD, and/or schizophrenia. Item-level GWAS revealed trait-specific genetic correlations with autism (items *r*_g_ range = − 0.27–0.78), ADHD (items *r*_g_ range = − 0.40–1), and schizophrenia (items *r*_g_ range = − 0.24–0.34). We find little evidence of common genetic liability across all neurodevelopmental traits but more so for several genetic factors across more specific areas of neurodevelopment, particularly social and communication traits. Some of these factors, such as one capturing prosocial behavior, overlap with factors found in the phenotypic analyses. Other areas, such as motor development, seemed to have more heterogenous etiology, with specific traits showing a less consistent pattern of genetic correlations with each other.

**Conclusions:**

These exploratory findings emphasize the etiological complexity of neurodevelopmental traits at this early age. In particular, diverse associations with neurodevelopmental conditions and genetic heterogeneity could inform follow-up work to identify shared and differentiating factors in the early manifestations of neurodevelopmental traits and their relation to autism and other neurodevelopmental conditions. This in turn could have implications for clinical screening tools and programs.

**Supplementary Information:**

The online version contains supplementary material available at 10.1186/s13229-024-00599-0.

## Introduction

Recent versions of international diagnostic classification systems have introduced an umbrella category of neurodevelopmental conditions. Conditions classified in this category typically manifest from childhood and are characterized by divergent trajectories of development. Generally, they are diagnosed based on significant difficulties in developmental skills in areas such as language, social abilities, learning, or motor activity. Neurodevelopmental conditions include autism spectrum conditions (autism) as well as conditions such as attention-deficit hyperactivity disorder (ADHD), intellectual disabilities, specific learning disabilities, developmental coordination disorder, and tic conditions. Some of these conditions had previously been conceptualized as independent and mutually exclusive conditions. For example, under DSM-IV, autism was an exclusion criterion for ADHD preventing their co-diagnosis. However, the recent shift towards their co-classification aligns diagnostic systems, such as the DSM, with a longstanding clinical awareness that observations of the specific traits and clinical features of neurodevelopmental conditions co-occur across diagnostic boundaries.

Neurodevelopmental conditions frequently co-occur [1, 2] and share symptoms at sub-diagnostic threshold levels [3, 4]. While the etiology of this co-occurrence is not well understood, some observations have implicated shared genetic liability between neurodevelopmental conditions. Unidentified latent genetic factors [5] as well as identified common [6–9] and rare genetic variants [10–12] are shared amongst many clinically-distinct neurodevelopmental conditions. Revisions of the formal diagnostic classification of neurodevelopmental conditions, such as those mentioned above, are in part a reflection of developments in our understanding of their common features [13–15]. However, it is also important to recognize that initial classifications were intended to describe characteristic symptom profiles rather than intended to imply inherent distinctions reflecting biological realities. That is, investigating the co-occurrence and shared etiology of different neurodevelopmental conditions can result in highly clinically-relevant insights without necessarily calling into question the distinctiveness and clinical utility of the conditions as separate entities.

Investigating the nosological and genetic bases for co-occurring neurodevelopmental conditions requires detailed data on their traits. Population-based registries, which collect diagnostic information from health care use for a given population, are typically limited to diagnostic (yes/no) outcomes. Clinical cohorts, which may have more detailed data, are generally smaller and commonly ascertain individuals based on a single condition. Thus, meaningful analyses of common genetic variants and shared etiology across areas of development and specific traits are difficult. Data collected in population-based cohorts, which are sampled from the general population and focused on longitudinal collection of data, typically have more breadth and depth of information that can help explore shared etiology of neurodevelopmental traits, but relatively fewer individuals with neurodevelopmental conditions. Still, relevant traits—capturing individual differences in language and motor development, attention, hyperactivity, social behavior, and repetitive, restricted behaviors and interests—can be observed in all children. These traits are likely influenced by some of the same underlying genetic liabilities as neurodevelopmental conditions [16–18]. The prospective nature of population-based birth cohorts means these traits can be studied early—prior to or around the age at which neurodevelopmental diagnoses are most commonly made [19, 20]. Exploring the relationships between neurodevelopmental traits early in life, investigating their genetic liabilities, and exploring links to neurodevelopmental conditions can give new insights into etiological mechanisms underlying the development and differentiation of such conditions.

Previous studies have examined the phenotypic factor structure of behaviors related to multiple neurodevelopmental conditions, primarily using items from questionnaires for both autism and ADHD in school-aged or older children. Out of five studies, four found differentiated dimensions of social communication, restricted and repetitive interests and behaviors, attention, and hyperactivity-impulsivity [21–24], while one found a common dimension for restricted and repetitive interests and behaviors with hyperactivity-impulsivity [25].

Regarding genetic factor structure, studies have found both shared and differentiating genetic factors between different domains of autism as well as with other neurodevelopmental conditions. Evidence from both twin and molecular genetic studies suggests that communication and repetitive interest and behavior traits of autism have genetically dissociable domains [26, 27]. Findings across a range of methodologies support correlated but separate genetic contributions to ADHD and autism [24, 28, 29]. Although the status and history of schizophrenia’s conceptualization as having neurodevelopmental origins is complex and warrants a more fulsome discussion [30, 31], it is worth noting that schizophrenia also shares genetic liability with both autism [7, 32, 33] and ADHD [29]. Initial evidence shows that this overlap contributes to different aspects of the phenotypic heterogeneity seen in autism [18, 27, 33]. Finally, across neurodevelopment more broadly, Pettersson et al. [5] found both a shared latent genetic factor across a range of different neurodevelopmental traits as well as specific genetic latent factors for impulsivity, learning problems, and autism and tics in a general population twin sample. In the present study, we leverage information on multiple traits related to different neurodevelopmental conditions. We investigate the phenotypic factor structure and genetic architecture underlying these early (age 3 years) neurodevelopmental traits in a large population-based birth cohort. We additionally investigate associations of these early signs with neurodevelopmental conditions at both the phenotypic and genotypic levels.

## Methods

### Measures and sample

#### Sample

The Norwegian Mother, Father and Child Cohort Study (MoBa) is a population-based pregnancy cohort study conducted by the Norwegian Institute of Public Health [34, 35]. Participants were recruited from all over Norway from 1999 to 2008. The women consented to participation in 41% of the pregnancies. Blood samples were obtained from both parents during pregnancy and from mothers and children (umbilical cord) at birth. The cohort includes approximately 114,500 children, 95,200 mothers and 75,200 fathers. The current study is based on version 12 of the quality-assured data files released for research in January 2019. The establishment of MoBa and initial data collection was based on a license from the Norwegian Data Protection Agency and approval from The Regional Committees for Medical and Health Research Ethics. The MoBa cohort is currently regulated by the Norwegian Health Registry Act. The current study was approved by The Regional Committees for Medical and Health Research Ethics (2016/1702).

The present study was conducted on a subset of the cohort (n = 58,630) who had information available from the 36-month questionnaire. The children were an average of 3.1 years (SD = 0.18) old when mothers completed the questionnaire. The sample had a 1.04:1 male-to- female ratio. Genetic analyses were conducted using a further quality controlled genotyped subset of the cohort (n = 42,934). For more information on the genotyping of the MoBa sample and for the family-based quality control pipeline used to prepare these data for analysis, see Corfield et al. [36].

#### Measures for neurodevelopmental traits

**Fig. 1:**
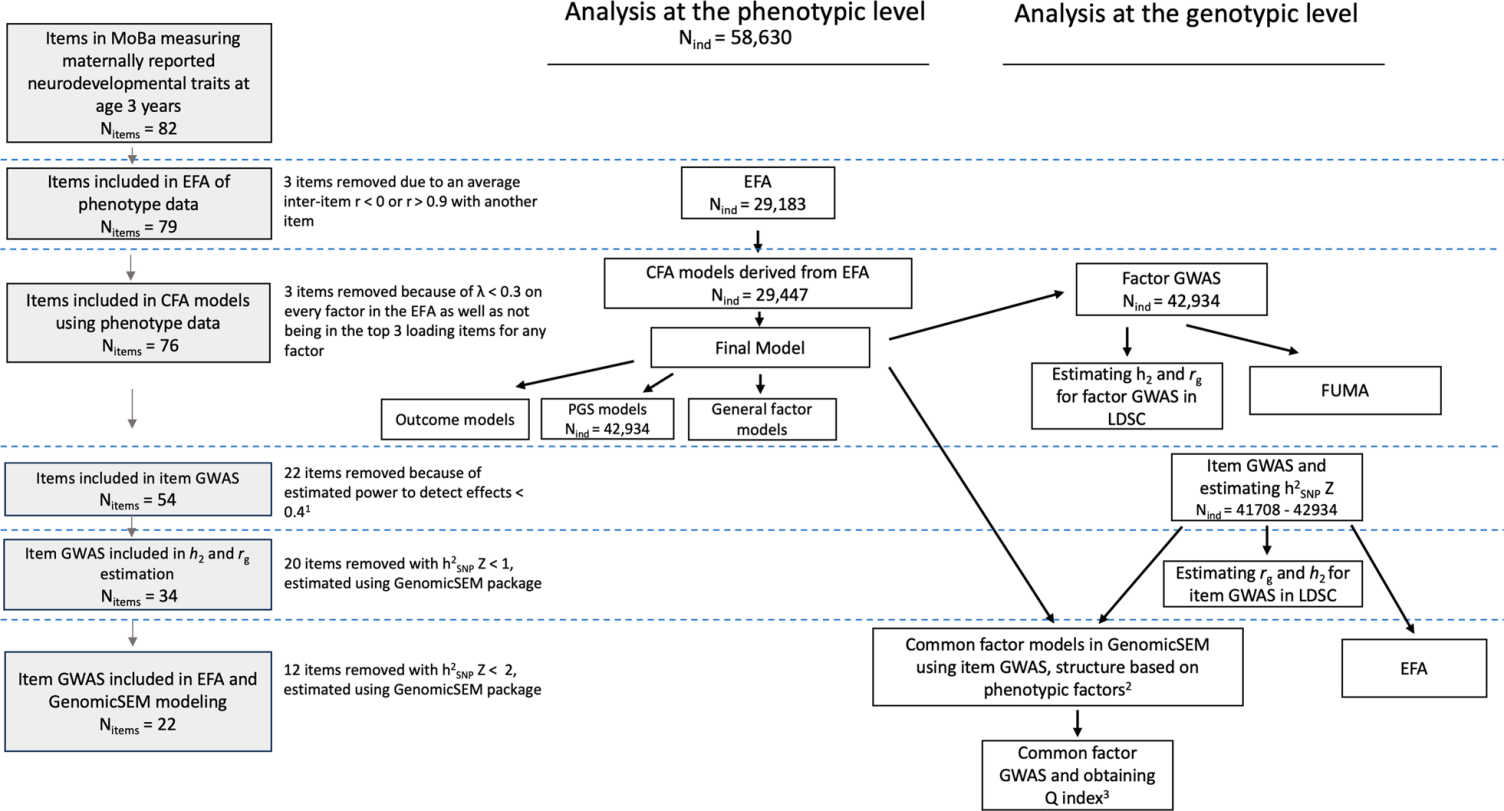
Outline of study design and main analyses at the phenotypic and genotypic levels. Grey boxes outline the steps where questionnaire items were removed with the exclusion thresholds listed to the right. Boxes indicate an analysis with the arrows denoting analyses which are based on (i.e., factor structure) or used results (i.e., summary statistics) from a previous analysis. Analyses conducted at the phenotypic level with no sample size listed were conducted in the full sample (N = 58,630). Half-samples for the EFA/CFA conducted in the phenotypic level were randomly selected halves of the full sample. Estimating rg refers to estimation of genetic correlations of the items/factors with neurodevelopmental conditions. 1 With the assumptions of an OR of 1.2, MAF of 0.01, and alpha of 0.01 in a logistic model with additive genetic effects. 2 Only common factor models with 3+ items run. 3 Common factor GWAS only run on models with good fits and significant factor loadings

We included items from all maternal report scales related to neurodevelopment in the 3-year questionnaire that asked about children’s observable behavior (as opposed to maternal concerns). Items were selected to cover areas of motor, language, social, communication, attention, activity regulation, sensory perception, and flexibility of behaviors and interests across multiple scales when possible (Fig. 1). This included items from the Social Communication Questionnaire (SCQ) [37], Ages and Stages Questionnaire (ASQ) [38], Non-Verbal Communication Checklist (NVCC) [39], Modified Checklist for Autism in Toddlers (M-CHAT) [40], Early Screening for Autistic Traits Questionnaire (ESAT) [41], the attention and hyperactivity subscale from the Child Behavior Checklist (CBCL) [42], the prosocial behaviors subscale of the Strength and Difficulties Questionnaire (SDQ) [43] as well as several MoBa-specific questions. All items included had either dichotomous (e.g., yes/no) or trichotomous (e.g., not true/sometimes true/often true) response categories. Items were reverse coded where needed so that higher values reflected greater endorsement of the trait.

#### Measures for diagnostic and clinically relevant outcomes

Diagnostic data was ascertained from the Norwegian Patient Register (NPR) between 2008 and June 2021 based on ICD-10 criteria using the R package *phenotools* [44]. Therefore, for the youngest in the cohort, diagnostic data was available from birth until approximately age 12, and for the oldest from approximately age 8 to 21. For those without diagnostic data from birth, the first diagnosis may be missing from registry data, but most will have the diagnostic code registered in subsequent healthcare use. Diagnostic groups were defined for receiving a diagnostic code at least one time for ADHD (F90), autism (F84.0, F84.1, F84.5, F84.8, and F84.9), intellectual disability and general developmental delay (F7 and F83), specific conditions of speech and language (F80, F98.5, F98.6), specific conditions of scholastic skills (F81), specific conditions of motor function (F82), and tic conditions (F95). The validity of autism diagnoses in MoBa has been studied previously [45]. Of 61 children identified with at least one instance of autism code in NPR, 58 (95%) received an autism diagnosis based on an independent multi-disciplinary standardized diagnostic assessment, and record review for another 567 children showed that for 86% the diagnostic criteria were well-documented in the health records [46]. No exclusions were made on the basis of other co-occurring disabilities or potentially contributing causes of disability (e.g., cerebral palsy, identified genetic syndromes, premature birth, birth complications) because this might lead to a biased or incomplete representation of children with neurodevelopmental conditions.

Sensitivity analyses for the main diagnostic outcomes (ADHD, autism, and intellectual disability/general developmental delay) were run restricting to individuals who had received a diagnostic code more than once to address the possibility of misdiagnosis or coding errors. Most diagnoses will have occurred after age 3, but some children will have already had a diagnosis at the time the questionnaire data was collected. This impacts different diagnostic outcomes differently. For example, using only individuals with NPR data available at age 3 so that all diagnoses by age 3 are captured, the percent of the diagnostic group who had received a diagnosis before age 4 was as low as 0.2% for ADHD, 6% for autism, 11% for intellectual disability/global developmental delay and up to 26% for specific conditions of motor function. Percentages for all outcomes are available in Additional file 1: Table S2.

Additionally, clinically relevant outcomes for having multiple neurodevelopmental conditions diagnoses registered as well as any psychiatric hospitalization were derived from NPR. Several clinically relevant outcomes were also coded using the MoBa questionnaire data. These included measures of maternal report of early (by age 3) referral to service use (habilitation service, educational psychology service, or child psychiatric clinic/department) as well as later maternally perceived impact and impairment from difficulties in development and behavior in their child’s life in the age 5 & 8 questionnaires. Further information on the scales, the items used in the factor models, and diagnostic and clinically relevant outcomes are available in Additional file 2:  supplementary methods and Additional file 1: Tables S1–3.

#### Polygenic scores

Polygenic scores (PGS) were estimated with the software PRSice2 [47] based on summary statistics from the most recent Psychiatric Genomic Consortium GWAS for ADHD [6], autism [7], and schizophrenia [48]. ADHD and autism were included as they are neurodevelopmental conditions with well powered and publicly available GWAS summary statistics. Schizophrenia was included given neurodevelopmental aspects to its development [31, 49, 50]. Scores were regressed on the first 10 genomic principal components (PCs) and genotype batch. The first principal component of 11 scores, constructed based on p-value thresholds between 5 × 10^–8^ and 1, was used for the subsequent analyses. This approach controls for type one error rate arising from optimization of pruning and thresholding while still maintaining prediction performance [51].

#### Analyses

An overview of the analyses performed as well as thresholds for item inclusion in each analytic step are presented in Fig. 1. Lenient thresholds for item selection were chosen to maximize the number of traits across different areas of development. Analytical code can be found at https://github.com/psychgen/neurodevelopment_traits_structure.

#### Exploratory and confirmatory factor analyses

Exploratory factor analysis (EFA) was performed in one randomly selected half of the full sample (n = 29,183). Confirmatory factor analyses (CFA) were run in the other half of the full sample (n = 29,447) for possible viable models derived from the EFA. Using standard fit indices (CFI, TLI, RMSEA) the best fitting model out of these possible models was used as the final model for all downstream analyses. In the full sample, both bifactor and higher-order models were run alongside the final selected correlated factor model to assess a unidimensional factor. To address potential sex differences in the measurement of these factors, we conducted measurement invariance testing in the full sample. A multi-group CFA (MG-CFA) of the correlated factor model by sex (N_males_ = 29,955, N_females_ = 28,589) was used to test for configural invariance and invariance of thresholds and loadings [52]. See Additional file 2: supplementary methods for further details on the factor analyses, criteria for model selection, and measurement invariance testing.

#### Measurement models with neurodevelopmental diagnoses, clinically relevant outcomes, and polygenic scores

The factor associations with diagnostic outcomes served two purposes:(1) validation and further characterization of the factors and (2) insight into how specific areas of development at age 3 are related to receiving a particular neurodevelopmental condition diagnosis. A correlated factor and a higher-order general factor model were run specifying the factors to predict neurodevelopmental diagnoses and other clinically relevant outcomes. In the correlated factor models, both univariate models with the factors predicting the outcomes individually and multiple regression models with factors predicting the outcome simultaneously were run. Due to collinearity concerns in the multiple regression models arising from groups of highly correlated factors, the magnitude of the factors’ effects within those groups were constrained to be equal in the correlated factor model. A higher-order model was run to assess if factors moderated the effect of a general factor on the outcomes as well as gain some insight into the factor effects on outcomes that are unique to the specific factor between highly correlated factors in the correlated factor model. In the higher-order model, general and specific factors were specified to predict outcomes separately in two models. Measurement models including PGS as explanatory variables for the factors were run in the correlated factors and higher-order model. Models were run in a multi-group SEM framework, grouped by sex with both regression effects and model parameters estimated for each sex separately.

#### Factor analyses software

EFA analyses were all run using the weighted least square mean and variance adjusted (WLSMV) estimation method and with a geomin oblique rotation applied in the Mplus statistical software (Muthén & Muthén, 2011). All CFA and measurement invariance models were run using the *lavaan* (v0.6–14) and *semTools* (v0.5–6) packages in R with the WLSMV estimation method [53, 54]. Missing data was handled using pairwise deletion for both the EFA and CFA, as it is the default in Mplus for categorical data.

#### Genome-wide association studies

Genome-wide association studies (GWAS) were run on each individual item (item GWAS) for which power calculations indicated sufficient statistical power, and on factor scores estimated for each factor (factor GWAS). This was done both to investigate the genetic effects underlying the factors we identified as well as to investigate the specificity of genetic effects between the factor and item levels of analysis. Factor scores were estimated using parameters for each sex from the correlated factor model multi-group CFA using the Empirical Bayes Model approach, the *lavaan* default method for categorical indicators. All GWAS included sex, genotype batch, and the first 10 PCs as covariates. Additional sex specific GWAS were run as sensitivity analyses for the factors. GWAS were run using version 3.1 of the REGENIE software, a computationally efficient linear mixed model method of conducting multi-trait GWAS. REGENIE can handle relatedness in the sample and correct for unbalanced case–control phenotypes in binary phenotypes [55]. For all factor and feasible item GWAS, SNP-based heritability (h^2^_SNP_) and genetic correlations (*r*_g_) with ADHD [6], autism [7], and schizophrenia [48] were estimated using linkage disequilibrium score regression (LDSC) [56]. Estimated h^2^_SNP_ for the item GWAS was on the liability scale. Functional mapping and annotation of the factor GWAS results were performed with FUMA (v1.5.3) [57]. Further information on sample sizes, prevalence estimates for LDSC, and power estimates used for the above analyses are listed in the Additional file 2: supplementary methods and Additional file 1: Table S4.

#### Genomic factor modeling and specificity of SNP effects

Genomic factor modeling used selected item GWAS. A lenient power inclusion threshold of Z > 2 as opposed to a more standard heuristic of Z > 4 for the item GWAS meant that power was borderline for genomic factor modeling. Because of this, an EFA was conducted on the estimated smoothed genetic correlation matrix of all chromosomes as opposed to only on even or odd chromosomes, which has been done to guard against overfitting if performing downstream analyses based on the EFA [9]. Therefore, no further downstream analyses (e.g., CFA) were conducted based on the results. Version 4.1.2 of the R package *stats* [58] was used to run the EFA and a promax rotation was applied. Common factor models based on factors from the phenotypic models that had at least three items meeting the item GWAS power threshold were run. For those with good fits and significant factor loadings, a common factor GWAS was run estimating SNP and Q_SNP_ effects. Q_SNP_ being a measure of how well the association of the SNP and the individual trait is accounted for by the factor [9, 59]. All confirmatory genomic factor modeling and GWAS were conducted using diagonally weighted least squares (DWLS) estimation in version 0.0.5 of the *GenomicSEM* R package [59].

## Results

### Phenotypic factor structure underlying early neurodevelopmental traits

Results of the EFA (Additional file 1: Tables S5–6) and CFA models indicated high dimensionality underlying early neurodevelopmental traits. Procedures to determine the optimal number of factors to retain indicated between 1 and 15 factors (Additional file 2: Figure S1) and fit indices from the EFA showed models with more than 9 factors met good fit criteria (Additional file 1: Table S5). Balancing these results with the interpretability of the factors, 3 models (9, 10, and 11-factor models) were selected to be run as confirmatory factor models in the other half of the sample. The 11-factor showed the best fit for complexity-penalized fit indices out of the three in both the EFA (Additional file 1: Table S5) and CFA (Additional file 1: Table S7). The 11-factor model was selected to be used in the downstream analyses.

**Fig. 2:**
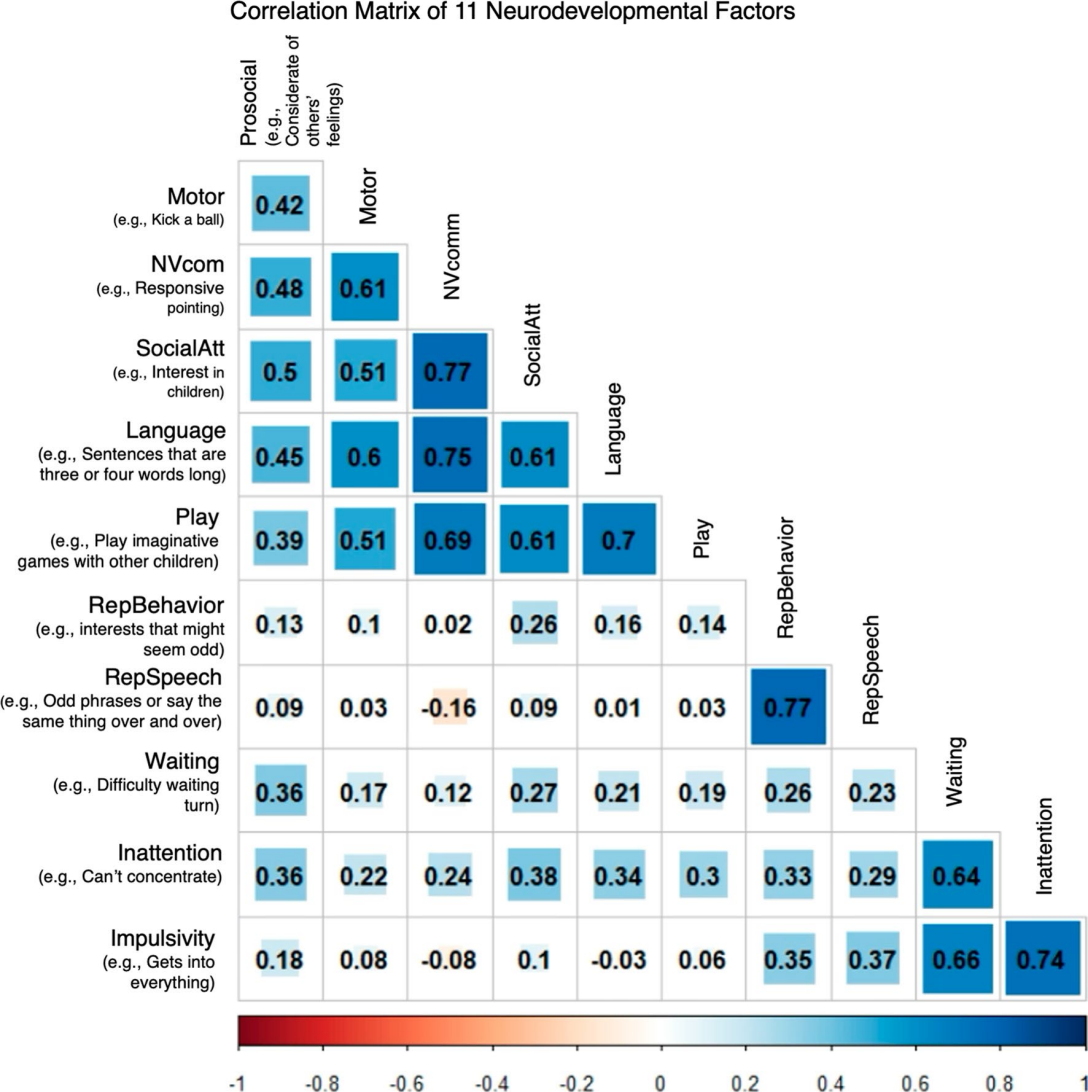
A correlation matrix of the 11 factors from the correlated factor model in the full population. Factors include prosocial behavior (prosocial), motor development (motor), nonverbal communication and joint attention (NVcom), social attention and interest (Social Att), language and verbal communication (language), play, repetitive and restricted behaviors and interests (RepBehavior), repetitive and idiosyncratic speech (RepSpeech), waiting, inattention and overactivity (inattention), and impulsivity. An example item from the factor is listed for each factor

The 11-factor model included factors roughly corresponding to areas of prosocial behavior (prosocial), motor development (motor), nonverbal communication and joint attention (NVcom), social attention and interest (SocialAtt), language and verbal communication (language), play, repetitive and restricted behaviors and interests (RepBehavior), repetitive and idiosyncratic speech (RepSpeech), waiting, inattention and overactivity (inattention), and impulsivity. Most items (73/76) loaded well (λ > 0.4) onto their respective factors (Additional file 2: Figure S2). Additionally, all factors except the idiosyncratic speech and impulsivity factors had moderate to high positive correlations with most other factors (Fig. 2). Factors covering the broad domains of social/communication, ADHD traits, and repetitive behaviors and speech were highly correlated amongst themselves but showed differing patterns of correlation with factors outside their broad domains. Parameter estimates of the final model are presented in Additional file 1: Tables S8–11. Finally, measurement invariance testing showed that invariance of thresholds and loadings held, so factors were assumed to largely represent the same constructs between males and females (Additional file 1: Table S12).

An additional general factor explaining all covariance between the different factors of early neurodevelopment had poor model fit indices (Hierarchical CFI: 0.621, TLI: 0.609, RMSEA: 0.032; Bifactor: CFI: 0.644, TLI: 0.624, RMSEA: 0.031) compared with the correlated factor model (CFI: 0.888, TLI: 0.883, RMSEA: 0.018) in the full sample. Besides fit indices, anomalous results in parameter estimates, non-uniform (λ = 0.07–0.89) loadings, and several specific factors with variances estimated close to zero indicated misspecification of the bifactor model to the data. For indicators other than model fit, this was less apparent in the hierarchical model (Additional file 1: Tables S13–14); therefore, it was used for further analyses. However, the general factor still exhibited varied loadings (λ = 0.313–0.787) and was characterized by factors encompassing social, communication, and motor development, which all had strong loadings from items with low endorsement in the general population.

### Factor validation and correlations with later outcomes

**Fig. 3:**
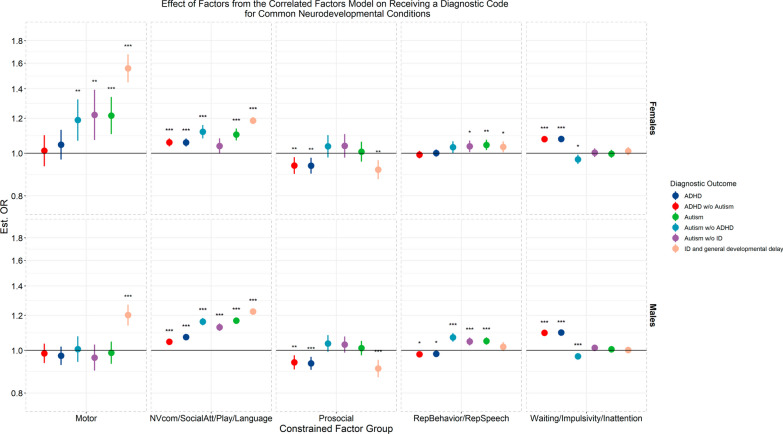
Estimated effects of factors from the correlated factor model in a multivariate regression controlling for the effects of all factors on the outcome for 5 selected diagnostic outcomes. Effects are presented as odds ratios calculated from the exponential of the standardized beta value from the logistic regression in the measurement models. 95% percent confidence intervals are shown. Due to high correlations amongst domains in the broad areas of social communication (the language & verbal communication, nonverbal communication and joint attention, play, and social attention and interest factors), ADHD-associated traits (the inattention and overactivity, waiting, impulsivity factors), and repetitive and restricted behaviors (the repetitive and idiosyncratic speech and repetitive and restricted behaviors and interests factors) effects of these factors were constrained to be equal to avoid collinearity issues. “*”, “**”, “***” denote adjusted *p* < 0.05, < 0.01, and < 0.001 respectively, after multiple testing correction. For full results of the outcome models, see the supplementary results (Additional file 2)

We found that nearly all early neurodevelopmental factors were associated with receiving a diagnosis of any of the neurodevelopmental conditions, higher perceived impact in daily life at ages 5 and 8, later psychiatric inpatient services, and reported early referral to habilitation, special education, and psychiatric services (Additional file 2: Figures S3–S5). In multiple regression models, all outcomes were still associated with at least one factor or group of highly correlated factors, and many were associated with multiple (Fig. 3; Additional file 2: Figures S6, S7). For example, both the highly correlated groups of the ADHD-trait factors, and social and communication factors were still associated with later receiving a diagnosis of ADHD. Estimates of these associations did not differ when restricting the sample to those who had received a diagnostic code at least twice, although precision decreased slightly (Additional file 2: Figure S8). Some of these associations also differed by sex, such as the motor factor being associated with an autism diagnosis only in girls in this model.

In the hierarchical model, where specific factors simultaneously predicted the outcomes, all factors were still associated with at least one outcome and some factors within the highly correlated factor groups had differing magnitude and direction of effects from each other (Additional file 2: Figures S9–S11). For example, out of the highly correlated social and communication factors, only the play and language factors were associated with ADHD. These two factors also had the most associations in the higher-order models, both being significantly associated with most of the diagnostic outcomes. The general factor was associated with all outcomes (Additional file 2: Figures S9–S11). However, the effect of a general factor on the outcome, when moderated by the specific factors, primarily explained additional variance in the outcomes related to early referral, general developmental delay/intellectual disability, and, in girls, specific language conditions when compared to the correlated factors model (Additional file 2: Figure S12).

### Common genetic variance underlying early neurodevelopmental traits

GWAS of the factor scores from the 11-factor model had low h^2^_SNP_ estimates. Four factors had estimated confidence intervals that crossed 0 (Additional file 1: Table S15). The highest estimate was the non-verbal communication factor (h^2^_SNP_ = 0.037 [0.013–0.061], *p* = 0.003). Four unique genome-wide significant loci identified across the factors, three of which were associated with multiple factors (Additional file 1: Table S16). Results from gene-based association analyses implemented in FUMA (significant *p* < 2.682 × 10^–6^; Table S17), identified *CNGB3* (*p* = 1.53 × 10^–6^) as associated with the motor factor as well as *RSRC1* (*p* = 3.95 × 10^–7^) and *ADAMTS17* (*p* = 8.19 × 10^–7^) with the prosocial behavior factor. Sex-stratified factor GWAS showed high genetic correlations with the factors in the full sample. These GWAS showed some differences in h^2^_SNP_ estimates by sex, but these differences did not reach statistical significance (Additional file 1: Table S18). 34 item GWAS reached our greater than 1 h^2^_SNP_ Z threshold (Additional file 1: Table S19), of which 21 items had h^2^_SNP_ that reached statistical significance. These items had a large range of estimated h^2^_SNP_ (range: 0.02–0.27; Additional file 1: Table S20) with differing levels of precision.

### Early neurodevelopmental traits relationships with genetic liability for neurodevelopmental conditions

**Fig. 4:**
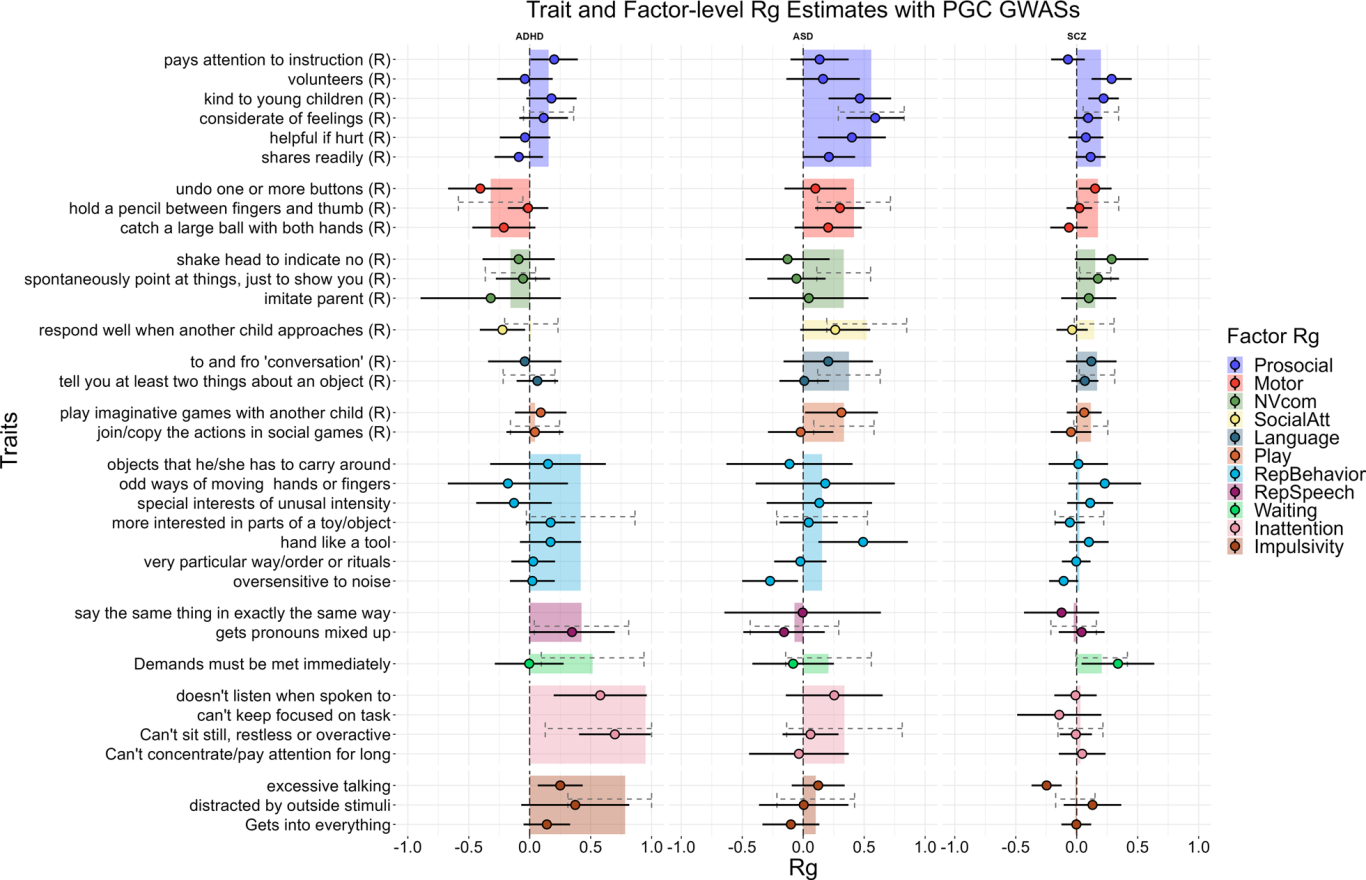
Estimated item and factor loading GWAS genetic correlation with PGS GWAS. 95% percent confidence intervals are presented. Results of multiple testing corrections are presented in Additional file 1: Tables S21 and S22 as a reference for the strength of statistical significance. Items are represented by points and factors are represented by bars. Bar width only reflects the number of items from the factor that were included. (R) denotes reversed coded items. The inattention factor had an estimated genetic correlation above one but is shown just below 1.0. This factor as well as the impulsivity factor had upper bounds of the confidence interval estimated over 1. Item-level estimates were removed if confidence intervals were estimated as having a range larger than 1.5

Genetic correlations between early neurodevelopmental traits and neurodevelopmental conditions were observed across multiple domains, as shown in Fig. 4, and were evident at both the factor (Additional file 1: Table S21) and item-level (Additional file 1: Table S22). ADHD had the highest genetic correlation with the inattentive and overactivity factor (*r*_g_ = 0.95 [0.13–1]). The prosocial behavior factor had the highest significant association for both autism (*r*_g_ = 0.56 [0.29–0.83]) and schizophrenia (*r*_g_ = 0.20 [0.05–0.34]). We find some instances of differing effects across conditions, such as the positive genetic correlation between the motor factor and autism (*r*_g_ = 0.42 [0.11–0.72]), and to a lesser extent, schizophrenia (*r*_g_ = 0.17 [0–0.34]) but a negative correlation with ADHD (*r*_g_ = − 0.32 [− 0.58 to − 0.01]).

The factors from the sex-stratified GWAS displayed similar genetic correlations with the neurodevelopmental conditions as in the entire sample but with slightly higher correlation estimates in males than females with autism and slightly higher in females than males with schizophrenia (Additional file 1: Table S23). These correlations were accompanied by large, overlapping confidence intervals but were in accordance with the findings of the PGS analyses. In these analyses, effects surviving multiple testing corrections were found exclusively in males for the autism PGS and in females for the schizophrenia PGS (Additional file 2: Figures S13–S14 and Additional file 1: Tables S24–S25).

At the item-level, the item “*considerate of feelings”* had the highest genetic correlation with autism, the item “*can’t sit still, restless or overactive”* with ADHD, and “*volunteers to help others”* with schizophrenia (Additional file 1: Table S22). A few item GWAS had differing effects compared to their specified factor’s GWAS. For example, the item measuring “*excessive talking”,* which was a part of the CBCL and loaded onto the impulsivity factor, was significantly negatively correlated (*r*_g_ = − 0.25 [− 0.37 to − 0.124]) with schizophrenia after multiple testing corrections while the impulsivity factor was uncorrelated with schizophrenia (*r*_g_ = − 0.01[− 0.17–0.15]).

### Genomic structure modeling and specificity of SNP effects

**Fig. 5:**
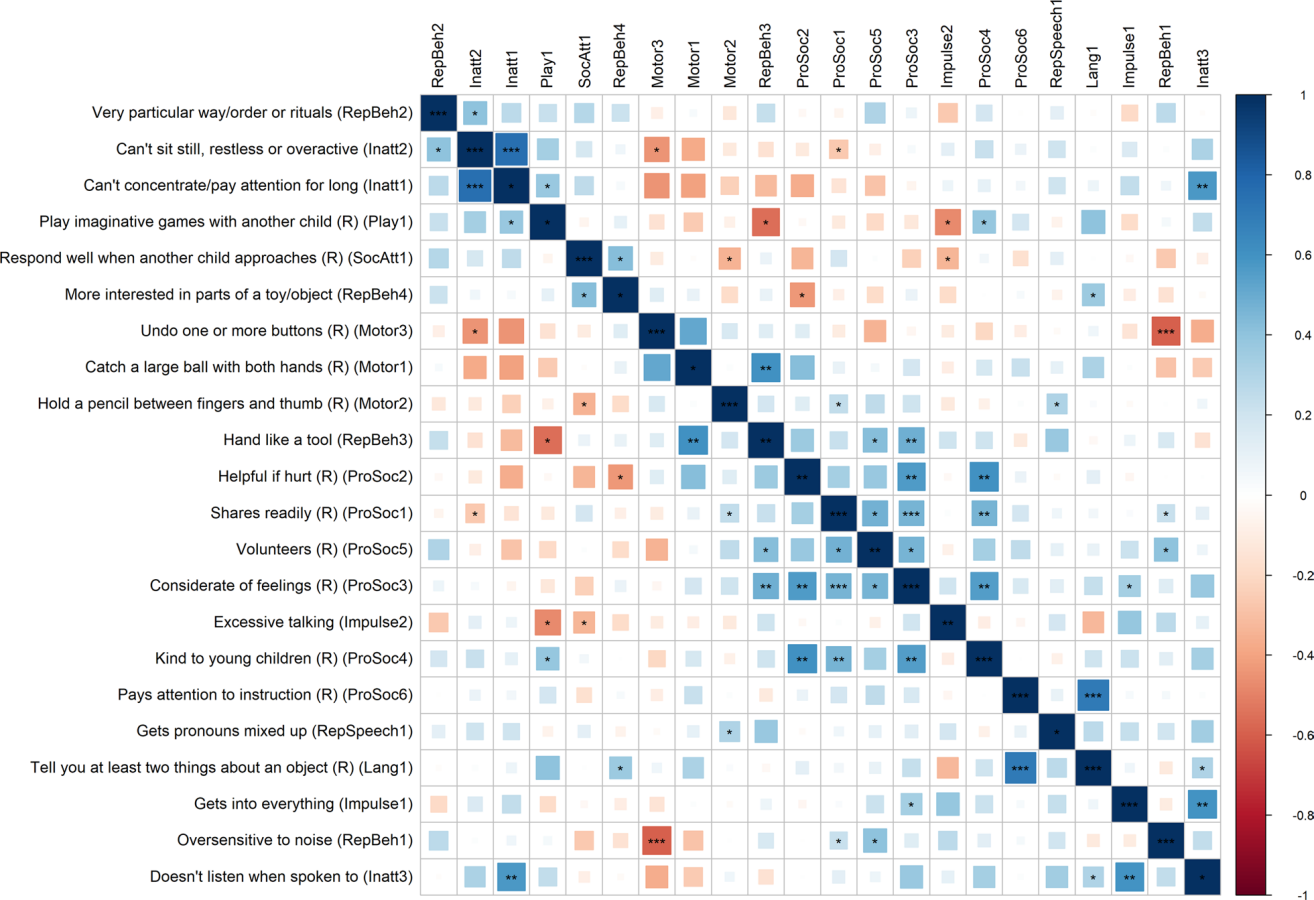
The estimated smoothed genetic correlations matrix for the 22 neurodevelopmental items used in the EFA and genetic factor modeling. Items order using angular order of the eigenvectors (AOE). "*”, “**”, “***” denote uncorrected *p* < 0.05, < 0.01, and < 0.001 respectively

Given power constraints, the EFA was run on the smoothed estimated genetic correlation matrix of all chromosomes and no further downstream analyses were performed. Genetic correlations between all items were estimated and are presented in Fig. 5. Two to three clusters of items seem to emerge from this, the most obvious being the prosocial behavior items and the item “*uses hand like a tool.*” These items were notably the items with the highest genetic correlations with autism. Extracting one “general” factor in the EFA left many items unrepresented. Further extractions of factors beyond a single factor were hard to interpret and frequently had factors defined by a few items, frequent cross-loadings, and strong negative loadings.

The motor, prosocial behavior, RepBehavior, and inattention factors were recreated via a CFA at the genomic level. Among these, only the prosocial behavior factor demonstrated an exceptional fit (CFI = 1, SRMR = 0.095; Additional file 2: Figure S15) and significant loadings for most items. The other common factor models either had loadings that did not reach statistical significance, fit indices that could not be estimated, or, in the case of the motor factor, did not converge. Based on this, we only performed a subsequent common factor GWAS for the prosocial behavior factor, which did not yield any genome-wide significant loci but identified more Q_snp_ than SNP hits at a suggestive association threshold (*p* < 5 × 10^–5^; see Additional file 2:supplementary results and Additional file 1: Tables S26–S27).

## Discussion

We leverage the item-level questionnaire data in up to 58,630 MoBa children to investigate patterns of relationships between specific traits from different areas of development in early childhood, the underlying genetic contributions, and potential shared etiology to clinically diagnosed neurodevelopmental conditions. We find that difficulties across all areas of early neurodevelopment are associated with receiving diagnoses across a range of neurodevelopmental conditions. Particularly, early difficulties in social and communication domains are associated with receiving a diagnosis of almost all neurodevelopmental conditions, suggesting that these are trans-diagnostically relevant in neurodevelopmental conditions. Additionally, the genetic contributions underlying variation in the general population for several early neurodevelopmental domains are genetically correlated with ADHD, autism, and schizophrenia. Finally, we find limited evidence of shared common genetic effects across all areas of neurodevelopment. From these results, we draw two over-arching conclusions. First, at both the phenotypic and genotypic levels, there is high heterogeneity in the underlying effects on variation in these traits—higher than would be expected if these traits were neatly aligned with distinct neurodevelopmental conditions. Secondly, despite their etiological and structural heterogeneity, early neurodevelopmental traits in a general population sample *are* phenotypically and genetically associated with neurodevelopmental diagnoses.

### Heterogeneity underlying early neurodevelopmental traits in a population-based sample

We find that most domains of neurodevelopment traits are at least moderately correlated with each other at the phenotypic level. The simplest etiological explanation for this would be shared liability across all areas of neurodevelopment, such as a general genetic neurodevelopment factor, which has been suggested based on a previous twin study [5]. However, we find substantial heterogeneity underlying early neurodevelopmental traits both at the phenotypic and genetic levels, and little evidence supporting a general factor of liability to all early neurodevelopmental traits at either level of analysis. Notwithstanding the question of the existence of a general factor for neurodevelopmental traits, we observe increased heterogeneity compared to what would be expected based on etiological factors that neatly lined up with diagnostic criteria. The observed factors are highly correlated amongst themselves in domains related to commonly separated neurodevelopmental domains (i.e., social and communication, repetitive behaviors, ADHD-related traits). However, these factors are differentially correlated with the factors outside of their domain and, when correlations between factors were accounted for by a general factor, have differing associations with later diagnoses. To note, while the neurodevelopmental traits are associated with and share some genetic variance with neurodevelopmental conditions, traits are non-specific to conditions. This pattern is consistent with co-occurrence between neurodevelopmental conditions being commonplace—in many cases "being the rule, rather than the exception" [3, 60, 61].

The results of the genomic factor modeling point towards a similar level of heterogeneity in the genetic architecture of early neurodevelopmental traits. We find some evidence for a genetic factor that resembles the prosocial factor identified in the phenotypic models and some shared genetic loci across areas of neurodevelopment, particularly across social and communication and prosocial behavior. However, in other areas, such as motor development, increased heterogeneity is observed at the item-level, potentially suggesting different genetic mechanisms underlying different aspects of motor skills. Lastly, even among the items measuring prosocial behavior, the higher number of Q_SNP_ hits compared to SNP hits contributing to the common genetic factor at a suggestive association threshold emphasizes the possibility of item-level specificity of genetic effects, even within the most coherent genetic factor.

### Early neurodevelopmental traits are associated with neurodevelopmental conditions

The factors identified as underpinning early neurodevelopmental traits in our sample were all associated with receiving a clinical diagnosis for different neurodevelopmental conditions. We find stronger associations between conditions and factors that contain items that overlap with diagnostic criteria of that condition, such as the inattention, impulsivity, and waiting factors with ADHD. One notable exception to this concerns the repetitive speech and behavior factors. Although these factors were independently associated with receiving an autism diagnosis, the effect sizes were lower compared to factors covering social communication difficulties. Further, when controlling for variation in other areas of development, these factors were no longer associated with receiving a diagnosis in some of the subgroups of the diagnostic outcome, such as girls who received an autism diagnosis but not a diagnosis for ADHD. What the repetitive speech and behavior factors are capturing in the general population should be considered in the interpretation of this finding. Most of the items that make up repetitive speech and behavior factors are from the SCQ, which, as a diagnostic screener, has mixed findings on the validity of its use for children under 4 [62]. Additionally, items in these factors are endorsed relatively frequently compared to the other SCQ items in our sample, meaning maternal reports of these items may be primarily capturing behaviors in the more typical range of these traits.

Stronger associations between factors and the diagnostic outcomes are seen for conditions that have higher rates of earlier referral or diagnosis in our sample, such as intellectual disability and specific motor conditions. The strength of these associations is likely impacted by the overlap in items with diagnostic criteria, however, we still find associations of factors with conditions with later average age of diagnosis, such as specific learning conditions, and with conditions that do not have diagnostic criteria overlapping with the factor, such as the social and communication factors with an ADHD diagnosis without co-occurring autism.

We find early childhood neurodevelopmental traits share common genetic liability with ADHD, autism, and schizophrenia. We identified associations between autism, and to a lesser extent, schizophrenia genetic liability with early motor, language, and social traits in contrast to some work in general populations, including in a smaller subset of MoBa, which have found few associations between these conditions and early childhood behaviors [17, 63]. Although the previous finding of autism genetic liability being associated with motor difficulties at age 3 in MoBa [17] remains in our larger sample. There are mixed findings on the association between ADHD genetic liability and social, communication, and repetitive behaviors and interests [64, 65]. We primarily find weak evidence for ADHD genetic liability contributing to early repetitive and idiosyncratic speech. Whilst it is tempting to read into discrepancies, it is important to note here the potential impact of well-characterized issues with the portability of polygenic scores across different study samples and populations [66], as well as the potential impact of chance variation—given the small effect sizes with which polygenic liability appears to manifest early in life [68]. Based on these inconsistent results across samples and  given the current stage of genomic discovery for neurodevelopmental conditions, this result should not be over-interpreted. However, our findings are in line with previous findings of inattention and hyperactivity traits as well as social and communication behavior in the general population sharing genetic variation with ADHD and autism, respectively [16, 65]. Finally, we observe other consistent findings with the literature, such as autism and schizophrenia genetic liability being associated with lower prosocial behavior [64, 67] and ADHD with fewer early motor difficulties [63].

Many items suggest similar associations to neurodevelopmental conditions as their factor. However, we do observe some trait-level heterogeneity  similar to the item-level or sub-domain level genomic analysis of neuroticism [69] and impulsivity [70]. For example, while the prosocial factor was genetically correlated with autism, only the factor’s items “*kind to young children*”, “*helpful when hurt*”, and “*considerate of feelings”* had associations with autism after multiple testing corrections. Items in the motor and repetitive behavior factors also show some trait-level heterogeneity. These observations offer some potential areas for follow-up work in clinical samples identifying differentiating mechanisms of early development across conditions. Our findings also identify some potential for shared mechanisms across domains at the sub-diagnostic level: for instance, repetitive behaviors and speech with ADHD traits. Genetic liability to ADHD has also been associated with repetitive behaviors and interests in clinical samples of autistic individuals [18, 33]. However, as previously mentioned, the potential impact of chance variation and the validity of items in a general population should also be considered with this observation. For example, items such as “*says the same thing over and over"* could be misinterpreted by parents, resulting in it capturing activity level or more common behaviors, rather than the idiosyncratic speech typically associated with autism.

### Limitations

There are some limitations of our study that should be considered. Despite splitting our sample into discovery and test halves, the exploratory factor analysis of such a diverse set of items in a large sample is likely to have led to some level of overfitting. Because of this, we do not suggest interpreting all identified factors as necessarily definitive distinct factors but instead put forward that there is increased dimensionality across areas of development with differing relationships to each other and neurodevelopmental conditions that may be lost at the diagnostic or scale level. Although we included measures of most behavioral domains of neurodevelopmental conditions, we did not have measures of all domains, such as cognitive ability and tics. Another consideration is that we used registry data to create our diagnostic outcomes. A limitation of this is that we cannot distinguish between subsequent co-occurring diagnoses and substitution diagnoses. Further, data being only available after 2008 means we do not know for certain how many had a diagnosis at the time mothers filled out the questionnaire. As these individuals were included this could impact the strength of the associations seen for some of the diagnostic outcomes.

Low power to detect signal for many of the item GWAS limits the claims. For the effects we identified the increased variation due to underpowered GWAS may contribute to the large range of estimated genetic correlations. However, power concerns are unlikely to fully explain the lack of a single general genetic factor. Our genetic analyses were also limited to common genetic variants, which may contribute to a lack of a general genetic factor as there is considerable overlap of rare variants associated with different neurodevelopmental conditions [10–12]. Finally, the genetic analyses were limited to participants in MoBa of European genetic ancestry, limiting the generalizability of our results across ancestries.

### Conclusions

Our exploratory results reveal the multidimensionality underlying early neurodevelopmental traits in a population-based birth cohort. These dimensions are broadly associated with receiving a diagnosis of neurodevelopmental conditions, and many are genetically correlated with ADHD, autism, and/or schizophrenia. We find little support for a shared common genetic liability across all traits in the general population. Instead, we observe multiple specific factors with certain shared genetic loci identified across, particularly, the social and communication domains of neurodevelopment, but none that are evidently relevant across all domains. Our trait-level analyses highlight the role of heterogenous genetic effects underlying early neurodevelopment traits and their relationships to neurodevelopmental conditions. These findings provide areas for further investigation to identify shared and distinct mechanisms across neurodevelopmental conditions.

### Supplementary Information

**Additional file 1:** Supplementary Tables.

**Additional file 2:** Supplementary Methods, Results, and Figures.

## Data Availability

The consent given by the participants does not allow for storage of data on an individual level in repositories. Researchers can apply for access to data for replication purposes via MoBa, in line with MoBa data access policies. Analytical code for this study can be found at https://github.com/psychgen/neurodevelopment_traits_structure. Documentation for MoBa questionnaires can be found at https://www.fhi.no/en/ch/studies/moba/for-forskere-artikler/questionnaires-from-moba/. Publicly available summary statistics provided by the Psychiatric Genomics Consortium (https://pgc.unc.edu/) were used for the creation of PGS and estimation of genetic correlations of autism, ADHD, and schizophrenia with the early neurodevelopmental traits.
